# Associations between education attainment and brain‐predicted age difference: a population‐based study

**DOI:** 10.1002/alz.089097

**Published:** 2025-01-09

**Authors:** Huijie Huang, Jiao Wang, Jun Ma, Weili Xu

**Affiliations:** ^1^ School of Public Health, Tianjin Medical University, Tianjin China; ^2^ Army Medical University, Chongqing, Chongqing China; ^3^ Aging Research Center, Karolinska Institutet, Stockholm Sweden

## Abstract

**Background:**

Education, as a proxy for cognitive reserves, has been linked to dementia; however, the association between education attainment and brain health remains unclear. We aim to investigate the association between education attainment and neuroimaging based brain‐predicted age difference (BPAD, the difference between brain age and chronological age).

**Method:**

The study included 32,322 chronic neurological disease‐free participants (mean age 54.74 ± 7.49 years, 46.58% female) who underwent brain MRI scans 9+ years after baseline. Years of education were self‐reported and categorized as low (7 and 10 years), medium (13, 15, and 19 years), and high (20 years) according to the International Standard Classification of Education. BPAD was predicted based on 1,079 multimodal imaging‐derived phenotypes (including structural and functional MRI) by a LASSO machine learning model. Alzheimer's disease‐related polygenic risk score (PRS_AD_) was tertiled as low, moderate and high. Data were analyzed using linear regressions.

**Result:**

At baseline, 6,332 (19.59%), 10,872 (33.64%), and 15,118 (46.77%) participants had low, medium, and high education, separately. Compared to low education, high education (β [95% CI]: ‐0.230 [‐0.392, ‐0.067]) was related to more negative BPAD. In joint‐effect analysis, the β (95% CI) of BPAD was ‐0.542 (‐0.796, ‐0.289) among participants with high education and low‐to‐moderate PRS_AD_, compared to those with low education and high PRS_AD_.

**Conclusion:**

High education attainment was associated with more negative BPAD, particularly among people with a low‐to‐moderate genetic susceptibility for dementia.

